# Alcohol abstinence and risk assessment for second esophageal cancer in Japanese men after mucosectomy for early esophageal cancer

**DOI:** 10.1371/journal.pone.0175182

**Published:** 2017-04-06

**Authors:** Akira Yokoyama, Chikatoshi Katada, Tetsuji Yokoyama, Tomonori Yano, Kazuhiro Kaneko, Ichiro Oda, Yuichi Shimizu, Hisashi Doyama, Tomoyuki Koike, Kohei Takizawa, Motohiro Hirao, Hiroyuki Okada, Takako Yoshii, Kazuo Konishi, Takenori Yamanouchi, Takashi Tsuda, Tai Omori, Nozomu Kobayashi, Haruhisa Suzuki, Satoshi Tanabe, Keisuke Hori, Norisuke Nakayama, Hirofumi Kawakubo, Hideki Ishikawa, Manabu Muto

**Affiliations:** 1 National Hospital Organization Kurihama Medical and Addiction Center, Kanagawa, Japan; 2 Department of Gastroenterology, Kitasato University School of Medicine, Kanagawa, Japan; 3 Department of Health Promotion, National Institute of Public Health, Saitama, Japan; 4 Department of Gastroenterology and Endoscopy, National Cancer Center Hospital East, Chiba, Japan; 5 Endoscopy Division, National Cancer Center Hospital, Tokyo, Japan; 6 Department of Gastroenterology, Hokkaido University Graduate School of Medicine, Kokkaido, Japan; 7 Department of Gastroenterology, Ishikawa Prefectural Central Hospital, Ishikawa, Japan; 8 Division of Gastroenterology, Tohoku University Graduate School of Medicine, Miyagi, Japan; 9 Division of Endoscopy, Shizuoka Cancer Center, Shizuoka, Japan; 10 Department of Surgery, National Hospital Organization Osaka National Hospital, Osaka, Japan; 11 Department of Gastroenterology and Hepatology, Okayama University Graduate School of Medicine, Dentistry and Pharmaceutical Sciences, Okayama, Japan; 12 Department of Gastroenterology, Kanagawa Cancer Center, Kanagawa, Japan; 13 Division of Gastroenterology, Department of Medicine, Showa University School of Medicine, Tokyo, Japan; 14 Department of Gastroenterology, Kumamoto Regional Medical Center, Kumamoto, Japan; 15 Department of Clinical Oncology, St. Marianna University School of Medicine, Kanagawa, Japan; 16 Endoscopy Center, Kawasaki Municipal Ida Hospital, Kanagawa, Japan; 17 Department of Diagnostic Imaging, Tochigi Cancer Center, Tochigi, Japan; 18 Research and Development Center for New Frontiers, Kitasato University School of Medicine, Kanagawa, Japan; 19 Department of Surgery, Keio University School of Medicine, Tokyo, Japan; 20 Department of Molecular-Targeting Cancer Prevention, Kyoto Prefectural University of Medicine, Kyoto, Japan; 21 Department of Therapeutic Oncology, Kyoto University Graduate School of Medicine, Kyoto, Japan; Baylor College of Medicine, UNITED STATES

## Abstract

**Background:**

Alcohol consumption combined with inactive aldehyde dehydrogenase-2 (ALDH2) and the presence of multiple esophageal Lugol-voiding lesions (LVLs; dysplasia) are strong predictors for multiple development of esophageal squamous cell carcinoma (ESCC) in East Asians. We invented a health risk appraisal (HRA) model for predicting the risk of ESCC based on drinking, smoking, dietary habits, and alcohol flushing, i.e., past or present facial flushing after drinking a glass of beer, a surrogate marker for inactive ALDH2.

**Methods:**

Prospective follow-up examinations (median follow-up time, 50.3 months) were performed in 278 Japanese men after endoscopic mucosectomy for early ESCC (UMIN Clinical Trials Registry ID: UMIN000001676).

**Results:**

Sixty-four subjects developed metachronous ESCC. A receiver operating characteristic curve showed that HRA scores ≥12 best predicted the development of metachronous ESCC. The ESCC detection rate per 100 person-years was 9.8 in the high-HRA-score group (n = 104) and 4.5 in the low-HRA-score group (n = 174), and the risk of development of metachronous ESCC was higher in the high-HRA-score group than in the low-HRA-score group (adjusted hazard ratio: 2.00 [95% CI: 1.12–3.30]). Multiple LVLs was a very strong predictor of the development of metachronous SCC, but high HRA scores predicted it independently. The cumulative incidences of metachronous ESCC decreased after drinking cessation in the high-HRA-score drinker group (adjusted hazard ratio: 0.37 [0.14–0.97]).

**Conclusions:**

Both the HRA model that included alcohol flushing and the multiple LVL grade predicted the development of metachronous ESCC in Japanese men after endoscopic mucosectomy for ESCC. Drinking cessation in the high-HRA-score drinker group reduced the rate of metachronous ESCC.

## Introduction

Technical improvements in endoscopes and a growing understanding of the endoscopic features of early squamous cell carcinoma (SCC) in the upper aerodigestive tract (UAT) have enabled early detection of SCC in the UAT [1,2]. Although treatment of early esophageal SCC by endoscopic mucosectomy has become a widespread practice in Japan and has succeeded in improving the outcome of this high-mortality cancer [3–5], follow-up endoscopic examinations have revealed an extremely high rate of development of metachronous SCC in the remaining esophagus and the head and neck [6–11]. Our recent prospective cohort study of patients who had undergone endoscopic mucosetcomy for early esophageal SCC demonstrated a strong association between the cumulative incidence of metachronous esophageal SCC and the grade of esophageal Lugol-voiding lesions (LVLs; squamous cell dysplasia) assessed by Lugol chromoendoscopy [12]. In addition, drinking cessation decreased the rate of developing metachronous esophageal SCC [12].

**Fig 1:**
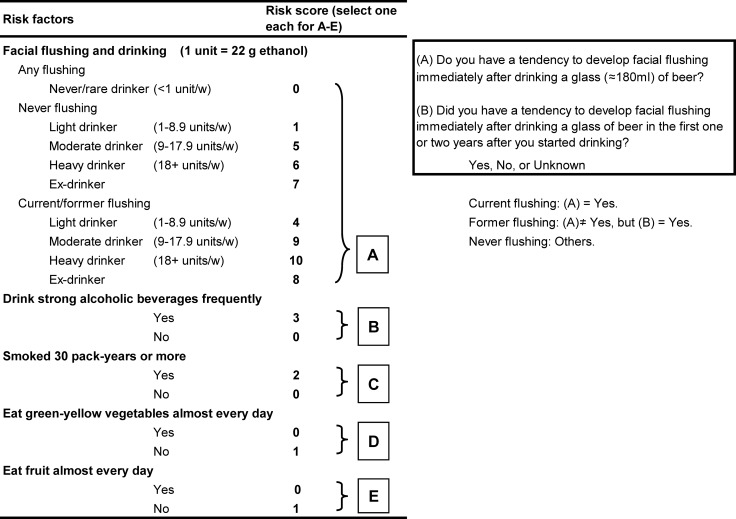
HRA model for predicting esophageal squamous cell carcinoma (SCC). The risk score is calculated as the sum of scores A to E. Higher scores mean higher risk of esophageal SCC.

Aldehyde dehydrogenase-2 (ALDH2) is a major enzyme in the metabolism of acetaldehyde after alcohol consumption, an established human carcinogen for SCC in the UAT [13]. A mutant allele (ALDH2*2; rs671) encodes an inactive subunit, and alcohol consumption by East Asians who are inactive ALDH2*2 carriers markedly increases their risk of SCC in the UAT [8,11,14–20]. We devised a simple alcohol flushing questionnaire for predicting the presence of inactive ALDH2 [21,22], and a health risk appraisal (HRA) model for esophageal SCC that consists of alcohol flushing and drinking, smoking, and dietary habits was developed based on the results of a Japanese male case-control study ([23]; Fig 1). In that study 58% of the esophageal SCC cases had HRA scores in the top 10% of the scores of the controls (an HRA score ≥11). Cross-sectional and follow-up mass-screening studies have shown very high esophageal SCC detection rates in groups with high-HRA-scores, i.e., scores ≥11 [24,25].

Higher risks of developing metachronous [8,11,12,26] and multiple [27–31] SCC in the UAT of Japanese subjects with esophageal SCC have been found to be associated with the presence of ALDH2*2, drinking, smoking, and lower fruit intake.

We therefore hypothesized that the risk of the first esophageal SCC assessed on the basis of the HRA score affected the risk of a second esophageal SCC after endoscopic mucosectomy for the first esophageal SCC.

The aim of this prospective study was to determine whether and to what extent the HRA score before the time of the first diagnosis of esophageal SCC alone or combined with the LVL grade predicts the development of metachronous esophageal SCC in male patients who have undergone endoscopic mucosectomy for early esophageal SCC. We also evaluated the effect of drinking cessation during the follow-up period combined with the initial HRA score on the development of metachronous esophageal SCC.

## Materials and methods

### Study design

From September 2005 to May 2010, we prospectively recruited patients of both sexes from 16 hospitals throughout Japan. This cohort study was approved by the institutional review board at each hospital; the Ethics Committee of National Hospital Organization Kurihama Medical and Addiction Center, the Ethics Committee of Kitasato University School of Medicine, the Ethics Committee of National Institute of Public Health, the Ethics Committee of National Cancer Center Hospital East, the Ethics Committee of National Cancer Center Hospital, the Ethics Committee of Hokkaido University Graduate School of Medicine, the Ethics Committee of Ishikawa Prefectural Central Hospital, the Ethics Committee of Tohoku University Graduate School of Medicine, the Ethics Committee of Shizuoka Cancer Center, the Ethics Committee of National Hospital Organization Osaka National Hospital, the Ethics Committee of Okayama University Graduate School of Medicine, Dentistry and Pharmaceutical Sciences, the Ethics Committee of Kanagawa Cancer Center, the Ethics Committee of Showa University School of Medicine, the Ethics Committee of Kumamoto Regional Medical Center, the Ethics Committee of St. Marianna University School of Medicine, the Ethics Committee of Kawasaki Municipal Hospital, the Ethics Committee of Tochigi Cancer Center, the Ethics Committee of Keio University School of Medicine, the Ethics Committee of Kyoto Prefectural University of Medicine, and the Ethics Committee of Kyoto University Graduate School of Medicine. We obtained a written informed consent from each patient (UMIN Clinical Trials Registry ID: UMIN000001676) [12].

### Study population

The referent cohort consisted of 332 patients of both sexes who had undergone complete endoscopic mucosectomy of early esophageal SCC. The inclusion criteria have been stated in an earlier report [12]. We selected the 278 male patients as the subjects of this study, because the HRA model evaluated in this study had been devised based on the results of an earlier study of esophageal SCC in men.

### LVL grading

LVLs were graded according to the maximum number of LVLs in at least one endoscopic field of view (A = no lesions; B = 1 to 9 lesions; C = ≥10 lesions). Endoscopic images obtained from the subjects at study entry were centrally reviewed in a blinded fashion by three endoscopists to grade the LVLs.

### HRA score

At the time of the baseline registration each subject was asked to fill out a structured questionnaire that asked questions concerning alcohol flushing response, drinking habits, smoking habits, and vegetable and fruit intake prior to the time of the first diagnosis of esophageal SCC. The questionnaire was the same as used in the earlier case-control study on which the HRA model was based [21,23]. The questionnaire asks whether the subject had a past or has a current tendency for facial flushing to occur after drinking a glass (≈ 180 ml) of beer, a surrogate marker of inactive ALDH2, and both the sensitivity and specificity of the flushing questionnaire as a means of identifying inactive ALDH2 in Japanese populations are approximately 90%. The HRA score was calculated as the sum of the scores A to E in Fig 1 which are logarithms of the multivariate odds ratio of each factor estimated in the previous case-control study.

### Follow-up examinations

Follow-up surveillance in the form of an endoscopic examination of the esophagus and Lugol chromoendoscopy was performed at 3-month intervals until 6 months after endoscopic mucosectomy of the initial esophageal SCC, and it was repeated every 6 months thereafter. If a new esophageal SCC was detected at the time of the first follow-up examination or thereafter, and was localized without continuity with the initial esophageal SCC, a diagnosis of “metachronous” SCC was made. SCC lesions present at registration as well as new lesions were centrally reviewed and confirmed in a blinded fashion by three pathologists.

### Alcohol and smoking cessation

At study entry, the physicians in charge handed a document describing the importance of cessation of drinking and smoking to all subjects and verbally instructed them to stop drinking and smoking. At each examination, we surveyed smoking status (non-smoker or smoker; and for smokers, the number of cigarettes smoked per day) and drinking status (nondrinker or drinker; and for drinkers, drinking frequency and amount of alcohol consumed) by self-report and instructed the subjects not to drink or smoke. The serum gamma-glutamyl transpeptidase (GGT) level was also measured at each examination.

### Statistical analysis

The data analysis in this study was performed 24 months after the endoscopic mucosectomy for the first esophageal SCC of the most-recently registered subject. Data are expressed as the mean ± SD or as a percentage. The geometric mean and geometric SE were calculated for GGT values that were log-normally distributed.A time-dependent receiver operating characteristic (ROC) curve at 24 months was used to assess the performance of the HRA model for predicting the development of metachronous esophageal SCC and to determine the optimal cut-off value [32]. Student’s *t*-test was used to compare the mean ages of different groups. *P* values for categorical data were calculated by using the Cochran-Mantel-Haenszel test for trends in LVL grade and Fisher’s exact test for other variables. The 95% confidence interval (95% CI) of the detection rate was estimated and tested based on a Poisson distribution. Cumulative percentages of patients with metachronous esophageal SCC were calculated according to the Kaplan-Meier method, and groups were compared by the log-rank test. The multivariate Cox proportional-hazards model was used to estimate the hazard ratio and 95% CI. All analyses were performed by using the SAS statistical package (version 9.1; SAS Institute, Cary, NC).

## Results

**Fig 2:**
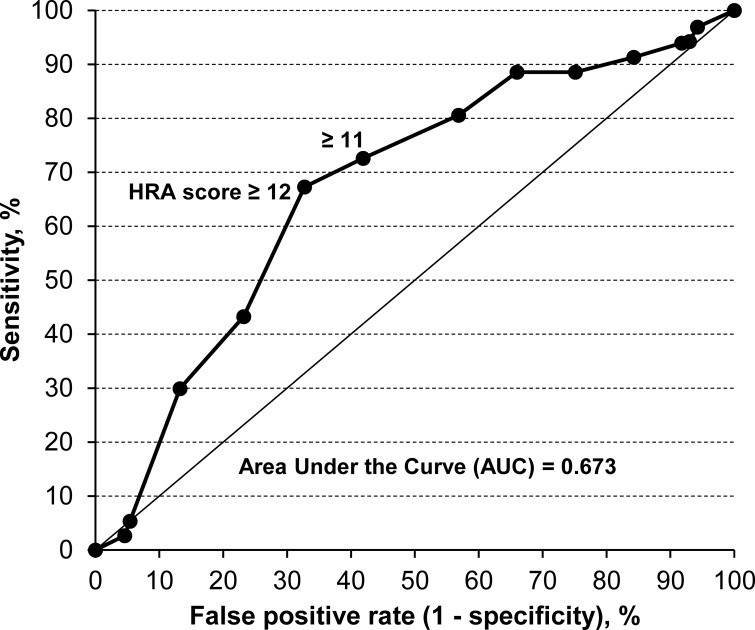
Time-dependent receiver operating characteristic curve according to HRA score at 24 months for predicting metachronous esophageal squamous cell carcinoma (SCC) in patients who had undergone endoscopic mucosectomy for early esophageal SCC.

The median follow-up period was 50.3 months (range, 1.3–81.2 months), and 64 subjects were diagnosed with metachronous esophageal SCC. Fig 2 shows the time-dependent ROC curve of the HRA model for predicting having developed metachronous esophageal SCC at 24 months, because the data analysis was performed 24 months after the endoscopic mucosectomy for the first esophageal SCC of the most-recently registered subject. The sensitivity of this possible predictor increased with a relatively small increase in the false-positive rate above the HRA score cutoff point of 12, at which the sensitivity was 67.3% and the false-positive rate was 32.7%. We therefore used an HRA score cutoff point of ≥12 in the following analyses.

HRA scores ≥12 selected 104 subjects (37.4%). Based on the cutoff points reported for mass-screening programs [23–25], HRA scores ≥11 selected 46.0% of the subjects, and when HRA scores were used as a cutoff point of 9 in the group aged 69 years and under and 8 in the group aged 70 years and over, 72.3% of the subjects were selected.

Table 1 summarizes the baseline characteristics of the subjects according to their HRA scores. The high-HRA-score group, i.e., group with scores ≥12, was characterized by heavier alcohol consumption, especially among current or former flushers, a greater preference for drinking strong alcoholic beverages straight, more cigarette smoking, and less intake of green and yellow vegetables and fruit. The LVL grade C was more common in the high-HRA-score group than in the low-HRA-score group. There were no significant differences in the proportions of abstainers from alcohol among those who had drunk 1 unit or more per day at baseline between the high- and low-HRA-score groups. The latest serum GGT levels of the abstainer group were significantly lower than in the non-abstainer group (geometric mean [geometric SE] = 26.5 [1.09] and 41.6 [1.08] IU/L, respectively, p<0.0001). There were no significant differences in the proportions of abstainers from tobacco among those who had smoked at baseline between the high- and low-HRA-score groups.

**Table 1:** Clinical characteristics of subjects according to HRA score.

		HRA score		
	Total	Low (0–11)	High (≥12)	P ^a^
	(n = 278)	(n = 174)	(n = 104)	
Age (years)				
40–59	16.9%	13.8%	22.1%	
60–69	45.3%	42.5%	50.0%	
70+	37.8%	43.7%	27.9%	0.019
Mean±SD	66.7±7.9	67.6±7.9	65.3±7.7	0.020
Current or former flushers	(n = 179)	(n = 85)	(n = 94)	
Never/rare drinkers ^b^	5.0%	10.6%	0.0%	
Light drinkers	19.0%	40.0%	0.0%	
Moderate drinkers	32.4%	31.8%	33.0%	
Heavy drinkers	33.0%	3.5%	59.6%	
Ex-drinker	10.6%	14.1%	7.4%	<0.0001
Never flushers	(n = 99)	(n = 89)	(n = 10)	
Never/rare drinkers ^b^	0.0%	0.0%	0.0%	
Light drinkers	12.1%	13.5%	0.0%	
Moderate drinkers	27.3%	30.3%	0.0%	
Heavy drinkers	52.5%	49.4%	80.0%	
Ex-drinker	8.1%	6.7%	20.0%	0.026
Strong alcoholic beverage consumption				
Frequent	11.9%	3.4%	26.0%	
Sometimes or never	88.1%	96.6%	74.0%	<0.0001
Smoking (pack years)				
<30	28.1%	37.9%	11.5%	
≥30	71.9%	62.1%	88.5%	<0.0001
Green-yellow vegetable consumption				
Seldom to 3–4 days/week	61.5%	50.0%	80.8%	
Almost every day	38.5%	50.0%	19.2%	<0.0001
Fruit consumption				
Seldom to 3–4 days/week	73.0%	63.2%	89.4%	
Almost every day	27.0%	36.8%	10.6%	<0.0001
LVL grade				
A	11.2%	13.2%	7.7%	
B	53.2%	55.7%	49.0%	
C	35.6%	31.0%	43.3%	0.025
Drinking cessation^c^	(n = 212)	(n = 117)	(n = 95)	
No	70.3%	73.5%	66.3%	
Yes	29.7%	26.5%	33.7%	0.29
Smoking cessation^d^	(n = 119)	(n = 58)	(n = 61)	
No	45.4%	41.4%	49.2%	
Yes	54.6%	58.6%	50.8%	0.46

HRA, health risk appraisal; LVL, Lugol-voiding lesion.

^a^ P-values are by t-test (mean age), Cochran-Mantel-Haenszel test for trend (LVL grade), or Fisher's exact test (other variables).

^b^ Never/rare, <1 unit/week; light, 1–8.9 units/week; moderate, 9–17.9 units/week; heavy, ≥18 units/week (1 unit = 22 grams ethanol).

^c^ Among those who had drunk 1 unit or more per day at baseline.

^d^ Among those who had smoked at baseline.

High HRA scores were associated with increases in the total number (95% CI) of metachronous esophageal SCC per 100 person-years (high-HRA-score group vs. low-HRA-score group; 9.8 [6.8–13.7] vs. 4.5 [3.0–6.5], p = 0.002; Table 2).

**Table 2:** Person-years and numbers of metachronous SCC in the esophagus according to HRA score.

		HRA score		
	Total	Low (0–11)	High (≥12)	P
Metachronous SCC	(n = 278)	(n = 174)	(n = 104)	
No. of events	64	29	35	
Person-years	998.3	642.2	356.1	
Per 100 person-years	6.4	4.5	9.8	0.002
(95% CI)	(4.9–8.2)	(3.0–6.5)	(6.8–13.7)	

HRA, health risk appraisal; SCC, squamous cell carcinoma.

**Fig 3:**
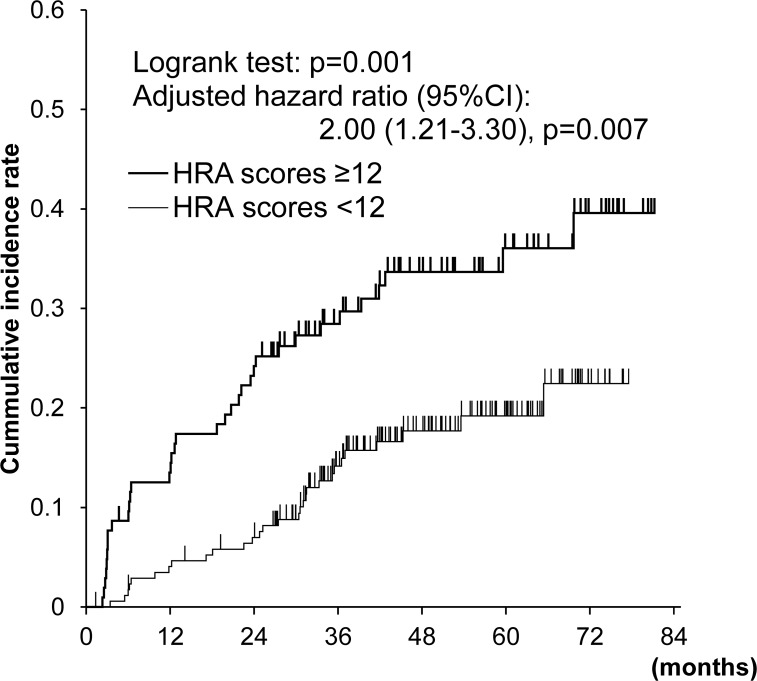
Cumulative incidences of metachronous esophageal squamous cell carcinoma in the esophagus according to HRA score. Hazard ratio was adjusted for age and LVL grades.

The cumulative incidences of metachronous esophageal SCC were significantly higher in the high-HRA-score group (p = 0.001; Fig 3).

When LVL grades and HRA scores were simultaneously entered into a multivariate Cox proportional-hazards model, LVL grade C was the strongest predictor of metachronous esophageal SCC (multivariate hazard ratio [95% CI]: 14.1 [1.93–103]; Table 3), but high HRA scores were an independent predictor of metachronous esophageal SCC (multivariate hazard ratio [95% CI] = 2.00 [1.21–3.30]). When LVL grades, HRA scores, and drinking cessation were simultaneously entered into the multivariate model for the group who had drunk 1 unit or more per day at baseline, drinking cessation was found to independently decrease the risk of metachronous esophageal SCC (multivariate hazard ratio [95% CI]: 0.45 [0.22–0.89]). Smoking cessation in the group who had smoked at baseline did not affect the risk of metachronous esophageal SCC.

**Table 3:** Prediction of metachronous SCC in the esophagus based on Cox proportional hazards models.

Metachronous SCC		Multivariate model 1 (n = 278)	Multivariate model 2 (n = 212)	Multivariate model 3 (n = 119)
		HR (95% CI) ^a^	HR (95% CI) ^a^	HR (95% CI) ^a^
LVL grade	A	1 (ref.)	0.00 (NC)	0.00 (NC)
	B	4.76 (0.64–35.3)	1 (ref.)	1 (ref.)
	C	14.1 (1.93–103)	3.23 (1.78–5.83)	4.17 (1.92–9.08)
	P for trend	<0.0001	<0.0001	
HRA score	Low (0–11)	1 (ref.)	1 (ref.)	1 (ref.)
	High (≥12)	2.00 (1.21–3.30)	2.17 (1.24–3.81)	1.91 (0.92–3.97)
	P value	0.007	0.007	0.085
Drinking cessation	No		1 (ref.)	
	Yes		0.45 (0.22–0.89)	
	P value		0.023	
Smoking cessation	No			1 (ref.)
	Yes			0.71 (0.35–1.45)
	P value			0.35

HR, hazard ratio; CI, confidence interval; LVL, Lugol-voiding lesion; SCC, squamous cell carcinoma.

NC, it was impossible to calculate the 95% CI because of the zero number of events in the LVL grade A group.

Model 1: all subjects.

Model 2: subjects who had drunk 1 unit or more per day at baseline.

Model 3: subjects who had smoked at baseline.

^a^ Grade of LVLs, HRA score, and drinking cessation (model 2) or smoking cessation (model 3) were simultaneously entered into a multivariate Cox proportional-hazards model; an adjustment was made for age but is not shown in this table.

**Fig 4:**
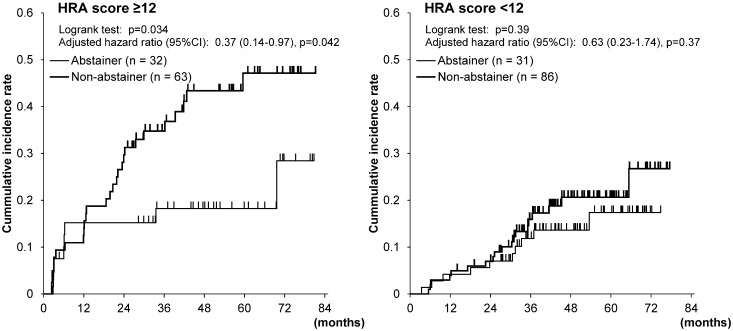
Cumulative incidences of metachronous esophageal squamous cell carcinoma in the esophagus according to drinking status in the high-HRA-score drinker group and in the-low-HRA-score drinker group. Hazard ratios were adjusted for age and LVL grades.

The cumulative incidences of metachronous esophageal SCC were markedly reduced by drinking cessation in the high-HRA-score group who had drunk 1 unit or more per day at baseline (Fig 4), and after adjustment for age and LVL grades the hazard ratio (95% CIs) for metachronous esophageal SCC was also reduced by drinking cessation (0.37 [0.14–0.97], p = 0.042) in this group. Drinking cessation among the low-HRA-score group did not affect the cumulative incidences of metachronous esophageal SCC.

## Discussion

The development of multiple SCCs in the UAT has been known as the phenomenon of “field cancerization” [33] and decreases the survival rate [26]. The results of follow-up studies of Japanese patients who had undergone endoscopic mucosectomy for early esophageal SCC have showed a very high prevalence of metachronous SCC in the UAT [6–11]. The results of the present prospective study showed that high HRA scores, i.e., ≥12, were associated with a two-fold higher risk of metachronous esophageal SCC. The esophageal SCC detection rate was much higher (9.8 per 100 person-years) in the high-HRA-score group. These results suggest that the risk of a first esophageal SCC assessed on the basis of the HRA scores to a considerable extent remains in regard to a second esophageal SCC.

As we have recently reported [12], the results of a present study showed that the highest LVL grade, i.e., grade C, was associated with a markedly high risk of metachronous esophageal SCC. High HRA scores ≥12 were positively associated with the LVL grade. However, the strong effect of high HRA scores on metachronous esophageal SCC was independent of the effect of LVL grades on metachronous esophageal SCC.

The HRA score was calculated based on the multivariate odds ratio of each risk factor estimated in the previous case-control study of esophageal SCC [15,21,23]. Calculating HRA scores makes it possible for people to identify their risk of esophageal SCC easily, and can be easily applied to public education or mass-screening. Follow-up chromoendoscopy was performed on 404 cancer-free controls in the original study, and the esophageal SCC detection rate per 100 person-years was 2.32 in the group with HRA scores ≥11 and 0.13 in the group with HRA scores <11 [24]. We applied the HRA questionnaire to endoscopic mass-screening of 2,221 Japanese men at 5 cancer screening facilities, and esophageal SCC was detected in 4.3% of the group with HRA scores ≥11, as opposed to 0.7% of the group with HRA scores <11 [25].

HRA scores ≥11 identified 58% of the esophageal SCC cases in the original case-control study [23], and in the present study HRA scores ≥11 identified 46.0% of the subjects. Since the HRA scores ≥11 have been found in 5–11% of Japanese male populations [23–25], the subjects of the present study had a very high background risk for esophageal SCC.

Previous Japanese studies have consistently demonstrated that the LVL grade C [6,7,9,10,27–29] and alcohol drinking with inactive heterozygous ALDH2 [8,11,27–31], which were associated with each other [20,27,29], were two strong risk factors for field cancerization in the UAT. Current or former flushing tendency after drinking a glass of beer, a surrogate marker of inactive ALDH2, identified inactive ALDH2 with 90% accuracy [21], and a very high risk of SCC in the UAT has been demonstrated in moderate or heavy drinkers who are current or former flushers [21,22,34]. Such high-risk drinkers accounted for 83.7% of the group with HRA scores ≥12 in the present study and 17.2% of the group with scores <12. Japanese studies have shown stronger associations between esophageal SCC and preference for drinking strong alcoholic beverages (whiskey and shochu) straight [15,34], which suggests the role of direct intact of high levels of acetaldehyde as an carcinogenic ingredient in whiskey and shochu [35]. There is substantial evidence that smoking and poor intake of green and yellow vegetables and fruit increase the risk of esophageal SCC [36,37]. All of the above risk factors were more common in the high-HRA-score group in this study, and that explains the good performance of the HRA score in predicting the risk of metachronous esophageal SCC.

We have reported that drinking cessation after endoscopic mucosectomy for early esophageal SCC substantially reduced the risk of metachronous SCC among drinkers, especially among those with LVL grade C [12]. A recent meta-analysis showed that the alcohol-related increased risk of esophageal cancer is reversed by alcohol cessation and the time-response relationship showed an exponential decay [38]. In the present study, drinking cessation in the high-HRA-score drinker group markedly reduced the risk of metachronous esophageal SCC. Informing high-risk persons of their high HRA scores will help persuade them to undergo follow-up examinations and change their lifestyle to prevent metachronous SCC. Smoking cessation did not significantly reduce the risk of metachronous esophageal SCC. Most epidemiologic studies have shown that the increased risk of esophageal SCC persisted for many years after smoking cessation and declined thereafter [39]. Drinking and smoking have a synergistic effect in increasing the risk of esophageal SCC [40], but drinking combined with alcohol flushing or inactive ALDH2 has an even greater effect [15, 17, 23]. It is conceivable that the very high prevalence of current or former flushers among the high-HRA-score drinkers contributed greatly to reducing their risk of metachronous esophageal SCC after drinking cessation. The time lag after which the risk reduction occurs and the magnitude of the reduction may partly account for the different effects of drinking cessation and smoking cessation. An investigation of the effectiveness of changing lifestyles is warranted.

Our follow-up study had several potential limitations. Although most of the subjects were followed-up for at least 24 months, and the median follow-up time was 50.3 months, there was wide individual variability in the follow-up period due to the 4.3-year subject registration period. The follow-up time variability may have limited the assessment of the relationship between the HRA scores and actual rate of metachronous SCC development. A longer term prospective study of the present cohort is currently under way. Recall bias and the reliability of self-reported drinking and smoking status are always problematic. However, we were able to confirm lower serum GGT levels in the abstainers. We excluded female patients from the referent population. ALDH2-related susceptibility to esophageal SCC has been demonstrated in Asian female drinkers [18,41,42]. A much smaller proportion of women who are heterozygous for ALDH2 than of men are drinkers [15,41]. Designing an HRA model for females will be the task of a future study.

In conclusion, the HRA model we devised for assessing the risk of esophageal SCC and the LVL grade were found to be good predictors of the development of metachronous esophageal SCC in Japanese men who had undergone endoscopic mucosectomy for early esophageal SCC. Drinking cessation in the high-HRA-score drinkers reduced the risk of metachronous esophageal SCC.

## References

[1] Muto M, Minashi K, Yano T, Saito Y, Oda I, Nonaka S, et al Early detection of superficial squamous cell carcinoma in the head and neck region and esophagus by narrow band imaging: a multicenter randomized controlled trial. J Clin Oncol. 2010; 28: 1566–1572. doi: 10.1200/JCO.2009.25.4680 20177025 10.1200/JCO.2009.25.4680 PMC2849774

[2] Katada C, Tanabe S, Koizumi W, Higuchi K, Sasaki T, Azuma M, et al Narrow band imaging for detecting superficial squamous cell carcinoma of the head and neck in patients with esophageal squamous cell carcinoma. Endoscopy. 2010; 42: 185–190. doi: 10.1055/s-0029-1243963 20195988 10.1055/s-0029-1243963

[3] Makuuchi H. Endoscopic mucosal resection for mucosal cancer in the esophagus. Gastrointest Endosc Clin N Am. 2001; 11: 445–458. 11778747

[4] Higuchi K, Tanabe S, Azuma M, Katada C, Sasaki T, Ishido K, et al A phase II study of endoscopic submucosal dissection for superficial esophageal neoplasms (KDOG 0901). Gastrointest Endosc. 2013; 78: 704–710. doi: 10.1016/j.gie.2013.04.182 23680178 10.1016/j.gie.2013.04.182

[5] Muto M, Satake H, Yano T, Minashi K, Hayashi R, Fujii S, et al Long-term outcome of transoral organ-preserving pharyngeal endoscopic resection for superficial pharyngeal cancer. Gastrointest Endosc. 2011; 74: 477–484. doi: 10.1016/j.gie.2011.04.027 21704994 10.1016/j.gie.2011.04.027

[6] Shimizu Y, Tsukagoshi H, Fujita M, Hosokawa M, Kato M, Asaka M. Metachronous squamous cell carcinoma of the esophagus arising after endoscopic mucosal resection. Gastrointest Endosc. 2001; 54: 190–194. 11474389 10.1067/mge.2001.116877

[7] Shimizu Y, Tsukagoshi H, Fujita M, Hosokawa M, Watanabe A, Kawabori S, et al Head and neck cancer arising after endoscopic mucosal resection for squamous cell carcinoma of the esophagus. Endoscopy. 2003; 35: 322–326. doi: 10.1055/s-2003-38151 12664389 10.1055/s-2003-38151

[8] Yokoyama A, Omori T, Yokoyama T, Sato Y, Kawakubo H, Maruyama K. Risk of metachronous squamous cell carcinoma in the upper aerodigestive tract of Japanese alcoholic men with esophageal squamous cell carcinoma: a long-term endoscopic follow-up study. Cancer Sci. 2008; 99: 1164–1171. doi: 10.1111/j.1349-7006.2008.00807.x 18429959 10.1111/j.1349-7006.2008.00807.x PMC11158932

[9] Urabe Y, Hiyama T, Tanaka S, Oka S, Yoshihara M, Arihiro K, et al Metachronous multiple esophageal squamous cell carcinomas and Lugol-voiding lesions after endoscopic mucosal resection. Endoscopy. 2009; 41: 304–309. doi: 10.1055/s-0029-1214477 19340732 10.1055/s-0029-1214477

[10] Hori K, Okada H, Kawahara Y, Takenaka R, Shimizu S, Ohno Y, et al Lugol-voiding lesions are an important risk factor for a second primary squamous cell carcinoma in patients with esophageal cancer or head and neck cancer. Am J Gastroenterol. 2011; 106: 858–866. doi: 10.1038/ajg.2010.489 21468010 10.1038/ajg.2010.489

[11] Kagemoto K, Urabe Y, Miwata T, Oka S, Ochi H, Kitadai Y, et al ADH1B and ALDH2 are associated with metachronous SCC after endoscopic submucosal dissection of esophageal squamous cell carcinoma. Cancer Med. 2016; 5: 1397–1404. doi: 10.1002/cam4.705 27038040 10.1002/cam4.705 PMC4944865

[12] Katada C, Yokoyama T, Yano T, Kaneko K, Oda I, Shimizu Y, et al Alcohol Consumption and Multiple Dysplastic Lesions Increase Risk of Squamous Cell Carcinoma in the Esophagus, Head, and Neck. Gastroenterology. 2016; 15: 860–869. e7. 10.1053/j.gastro.2016.07.040 27492616

[13] International Agency for Research on Cancer. IARC Monographs on the Evaluation of Carcinogenic Risks to Humans Vol. 96 Alcohol beverage consumption and ethyl carbamate (urethane). IARC, Lyon, 2010. PMC4781168 21735939

[14] Yokoyama A, Muramatsu T, Ohmori T, Higuchi S, Hayashida M, Ishii H. Esophageal cancer and aldehyde dehydrogenase-2 genotypes in Japanese males. Cancer Epidemiol Biomarkers Prev. 1996; 5: 99–102. 8850269

[15] Yokoyama A, Kato H, Yokoyama T, Tsujinaka T, Muto M, Omori T, et al Genetic polymorphisms of alcohol and aldehyde dehydrogenases and glutathione S-transferase M1 and drinking, smoking, and diet in Japanese men with esophageal squamous cell carcinoma. Carcinogenesis. 2002; 23: 1851–1859. 12419833 10.1093/carcin/23.11.1851

[16] Boccia S, Hashibe M, Galli P, De Feo E, Asakage T, Hashimoto T, et al Aldehyde dehydrogenase 2 and head and neck cancer: a meta-analysis implementing a Mendlian randomization approach. Cancer Epidemiol Biomarkers Prev. 2009; 18: 248–254. doi: 10.1158/1055-9965.EPI-08-0462 19124505 10.1158/1055-9965.EPI-08-0462

[17] Cui R, Kamatani Y, Takahashi A, Usami M, Hosono N, Kawaguchi T, et al Functional variants in ADH1B and ALDH2 coupled with alcohol and smoking synergistically enhance esophageal cancer risk. Gastroenterology. 2009; 137: 1768–1775. doi: 10.1053/j.gastro.2009.07.070 19698717 10.1053/j.gastro.2009.07.070

[18] Yang SJ, Yokoyama A, Yokoyama T, Huang YC, Wu SY, Shao Y, et al Relationship between genetic polymorphisms of ALDH2 and ADH1B and esophageal cancer risk: A meta-analysis. World J Gastroenterol. 2010; 16: 4210–4220. doi: 10.3748/wjg.v16.i33.4210 20806441 10.3748/wjg.v16.i33.4210 PMC2932928

[19] Tanaka F, Yamamoto K, Suzuki S, Inoue H, Tsurumaru M, Kajiyama Y, et al Strong interaction between the effects of alcohol consumption and smoking on oesophageal squamous cell carcinoma among individuals with ADH1B and/or ALDH2 risk alleles. Gut. 2010; 59: 1457–1464. doi: 10.1136/gut.2009.205724 20833657 10.1136/gut.2009.205724

[20] Yokoyama A, Hirota T, Omori T, Yokoyama T, Kawakubo H, Matsui T, et al Development of squamous neoplasia in esophageal iodine-unstained lesions and the alcohol and aldehyde dehydrogenase genotypes of Japanese alcoholic men. Int J Cancer. 2012; 130: 2949–2960. doi: 10.1002/ijc.26296 21796615 10.1002/ijc.26296

[21] Yokoyama T, Yokoyama A, Kato H, Tsujinaka T, Muto M, Omori T, et al Alcohol flushing, alcohol and aldehyde dehydrogenase genotypes, and risk for esophageal squamous cell carcinoma in Japanese men. Cancer Epidemiol Biomarkers Prev. 2003; 12: 1227–1233. 14652286

[22] Brooks PJ, Enoch MA, Doldman D, Li TK, Yokoyama A. The alcohol flushing response: an unrecognized risk factor of esophageal cancer from alcohol consumption, PLoS Med. 2009; 6: e50 doi: 10.1371/journal.pmed.1000050 19320537 10.1371/journal.pmed.1000050 PMC2659709

[23] Yokoyama T, Yokoyama A, Kumagai Y, Omori T, Kato H, Igaki H, et al Health Risk Appraisal Models for Mass Screening of Esophageal Cancer in Japanese Men. Cancer Epidemiol Biomarkers Prev. 2008; 17: 2846–2854. doi: 10.1158/1055-9965.EPI-08-0397 18843030 10.1158/1055-9965.EPI-08-0397

[24] Yokoyama A, Kumagai Y, Yokoyama T, Omori T, Kato H, Igaki H, et al Health risk appraisal models for mass screening for esophageal and pharyngeal cancer: an endoscopic follow-up study of cancer-free Japanese men. Cancer Epidemiol Biomarkers Prev. 2009; 18: 651–655. doi: 10.1158/1055-9965.EPI-08-0758 19190142 10.1158/1055-9965.EPI-08-0758

[25] Yokoyama A, Oda J, Iriguchi Y, Kumagai Y, Okamura Y, Matsuoka M, et al A health-risk appraisal model and endoscopic mass screening for esophageal cancer in Japanese men. Dis Esophagus. 2013; 26: 148–153. doi: 10.1111/j.1442-2050.2012.01343.x 22458712 10.1111/j.1442-2050.2012.01343.x

[26] Matsubara T, Yamada K, Nakagawa A. Risk of second primary malignancy after esophagectomy for squamous cell carcinoma of the thoracic esophagus. J Clin Oncol. 2003; 21: 4336–4341. doi: 10.1200/JCO.2003.12.074 14645422 10.1200/JCO.2003.12.074

[27] Muto M, Hitomi Y, Ohtsu A, Ebihara S, Yoshida S, Esumi H. Association of aldehyde dehydrogenase 2 gene polymorphism with multiple oesophageal dysplasia in head and neck cancer patients. Gut. 2000; 47: 256–261. doi: 10.1136/gut.47.2.256 10896918 10.1136/gut.47.2.256 PMC1727996

[28] Muto M, Takahashi M, Ohtsu A, Ebihara S, Yoshida S, Esumi H. Risk of multiple squamous cell carcinomas both in the esophagus and the head and neck region. Carcinogenesis. 2005; 26: 1008–1012. doi: 10.1093/carcin/bgi035 15718256 10.1093/carcin/bgi035

[29] Katada C, Muto M, Nakayama M, Tanabe S, Higuchi K, Sasaki T, et al Risk of superficial squamous cell carcinoma developing in the head and neck region in patients with esophageal squamous cell carcinoma. Laryngoscope. 2012; 122: 1291–1296. doi: 10.1002/lary.23249 22674532 10.1002/lary.23249

[30] Yokoyama A, Muramatsu T, Ohmori T, Makuuchi H, Higuchi S, Matsushita S, et al Multiple primary esophageal and concurrent upper aerodigestive tract cancer and the aldehyde dehydrogenase-2 genotype of Japanese alcoholics. Cancer. 1996; 77: 1986–1990. doi: 10.1002/(SICI)1097-0142(19960515)77:10<1986::AID-CNCR4>3.0.CO;2-F 8640660 10.1002/(SICI)1097-0142(19960515)77:10<1986::AID-CNCR4>3.0.CO;2-F

[31] Yokoyama A, Watanabe H, Fukuda H, Haneda T, Kato H, Yokoyama T, et al Multiple cancers associated with esophageal and oropharyngolaryngeal squamous cell carcinoma and the aldehyde dehydrogenase-2 genotype in male Japanese drinkers. Cancer Epidemiol Biomarkers Prev. 2002; 11: 895–900. 12223435

[32] Heagerty PJ, Lumley T, Pepe MS. Time-dependent ROC curves for censored survival data and a diagnostic marker. Biometrics. 2000; 56: 337–344. 10877287 10.1111/j.0006-341x.2000.00337.x

[33] Slaughter DP, Southwick HW, Smejkal W. ‘‘Field cancerization” in oral stratified epithelium. Cancer. 1953; 6: 963–968. 13094644 10.1002/1097-0142(195309)6:5<963::aid-cncr2820060515>3.0.co;2-q

[34] Asakage T, Yokoyama A, Haneda T, Yamazaki M, Muto M, Yokoyama T, et al Genetic polymorphisms of alcohol and aldehyde dehydrogenases, and drinking, smoking and diet in Japanese men with oral and pharyngeal squamous cell carcinoma. Carcinogenesis. 2007; 28: 865–874. doi: 10.1093/carcin/bgl206 17071628 10.1093/carcin/bgl206

[35] Lachenmeier DW, Sohnius EM. The role of acetaldehyde outside ethanol metabolism in the carcinogenicity of alcoholic beverages: evidence from a large chemical survey. Food Chem Toxicol. 2008; 46: 2903–2911. doi: 10.1016/j.fct.2008.05.034 18577414 10.1016/j.fct.2008.05.034

[36] Yamaji T, Inoue M, Sasazuki S, Iwasaki M, Kurahashi N, Shimazu T, et al Fruit and vegetable consumption and squamous cell carcinoma of the esophagus in Japan: the JPHC study. Int J Cancer. 2008; 123:1935–1940. doi: 10.1002/ijc.23744 18688852 10.1002/ijc.23744

[37] Jin J, Ouyang Z, Wang Z. Association of fruit and vegetables with the risk of nasopharyngeal cancer: evidence from a meta-analysis. Sci Rep. 2014; 4: 5229 doi: 10.1038/srep05229 25008797 10.1038/srep05229 PMC5381608

[38] Jarl J, Gerdtham UG. Time pattern of reduction in risk of oesophageal cancer following alcohol cessation–a meta-analysis. Addiction. 2012; 107: 1234–1243. doi: 10.1111/j.1360-0443.2011.03772.x 22175692 10.1111/j.1360-0443.2011.03772.x

[39] Bosetti C, Gallus S, Garavello W, La Vecchia C. Smoking cessation and the risk of oesophageal cancer: An overview of published studies. Oral Oncol. 2006; 42: 957–964. doi: 10.1016/j.oraloncology.2006.03.007 16919996 10.1016/j.oraloncology.2006.03.007

[40] Morita M, Kumashiro R, Kubo N, Nakashima Y, Yoshida R, Yoshinaga K, et al Alcohol drinking, cigarette smoking, and the development of squamous cell carcinoma of the esophagus: epidemiology, clinical findings, and prevention. Int J Clin Oncol. 2010; 15: 126–134. doi: 10.1007/s10147-010-0056-7 20224884 10.1007/s10147-010-0056-7

[41] Yokoyama A, Kato H, Yokoyama T, Igaki H, Tsujinaka T, Muto M, et al Esophageal squamous cell carcinoma and aldehyde dehydrogenase-2 genotypes in Japanese females. Alcohol Clin Exp Res. 2006; 30: 491–500. doi: 10.1111/j.1530-0277.2006.00053.x 16499490 10.1111/j.1530-0277.2006.00053.x

[42] Wang Y, Ji R, Wei X, Gu L, Chen L, Rong Y, et al Esophageal squamous cell carcinoma and ALDH2 and ADH1B polymorphisms in Chinese females. Asian Pac J Cancer Prev. 2011; 12: 2065–2068. 22292652

